# The complete mitochondrial genome of *Cryptotermes declivis* Tsai et Chen (Isoptera: Kalotermitidae)

**DOI:** 10.1080/23802359.2019.1627950

**Published:** 2019-07-16

**Authors:** Peishan He, Ling Zhang, Yang Wen, Ting Li, Kaiping Hu, Quan Zhou, Jianguo Wang

**Affiliations:** School of Agricultural Sciences, Jiangxi Agricultural University, Nanchang, P. R. China

**Keywords:** *Cryptotermes declivis*, mitochondrial genome, phylogenetic tree

## Abstract

The complete mitochondrial genome (mitogenomes) of *Cryptotermes declivis* were revealed. The circular mitogenome of *C. declivis* (accession number MK599465) has a length of 15678 bp and an overall G + C content of 35.07%. Genome orientation showed rearrangement. The position of the *trnV* is moved from the L-strand between *rrnL* and *rrnS* to the H-strand between *rrnS* and *trnI*. The data further expands arthropod mitogenome databases and provides a case of gene rearrangement.

*Cryptotermes declivis* (Isoptera: Kalotermitidae) is the most common *Cryptotermes* species in China (Tang 2015). It is an important pest affecting wooden structures. In this study, *C. declivis* specimens were collected in Maoming, Guangdong Province, China. The voucher samples were kept at the Laboratory of Invasion Biology, College of Agriculture, Jiangxi Agricultural University, Jiangxi, China.

The complete mitochondrial genome of *C. declivis* (Genbank: MK599465) revealed the size of 15,678 bp, containing A 41.37%, G 13.03%, T 23.56%, and C 22.04%. It is highly A + T biased, accounting for 64.93%. The *C. declivis* mitogenome content 13 protein-coding genes (PCGs), 22 transfer RNA (tRNA) genes, and 2 ribosomal RNA (rRNA) genes. The nucleotide sequence of 13 PCGs of all mitochondrial genes was 11,081 bp in length. The sizes of 2 rRNA genes were 1384 bp and 808 bp, respectively. All of the 22 tRNAs, ranging from 61 to75 bp, have a classical cloverleaf structure, except for *trnR* lacking the TψC loop and *trnS1* lacking the DHU arm and loop.

Except for *nad5*, *nad4*, *nad4L*, *nad1*, *rrnL*, *rrnS* gene, and seven tRNA genes (*trnQ*, *trnC*, *trnY*, *trnF*, *trnH*, *trnP*, *trnL1*) encoded on the Light Strand (L-strand), the remaining genes were encoded on the heavy strand (H-strand). This gene order is not arranged as the putative ancestral insect mitochondrial genome ( Bourguignon et al. 2015 ). This species showed rearrangement in the mitochondrial genome. The position of the *trnV* is moved from the L-strand between *rrnL* and *rrnS* to the H-strand between *rrnS* and *trnI*.

The MrBayes molecular phylogeny in Figure 1 is based on the 13 PCGs and 24 RNAs (22tRNAs, 2RNAs), using PhyloSuite (Ronquist et al. 2012; Katoh and Standley 2013; Lanfear et al. 2016; Zhang et al. 2018). This result was similar to the previous molecular studies of Kalotermitidae (Liao et al. 2018).

**Figure 1.**
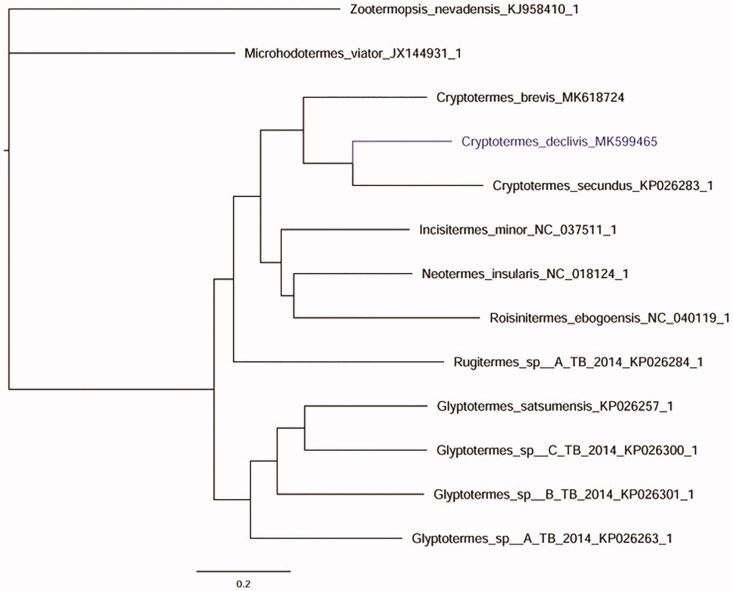
MrBayes phylogenetic tree based on the 13 PCGs and 24 RNAs (22tRNAs, 2rRNAs) of Kalotermitidae termite mitogenomes. *Microhodotermes viator* and *Zootermopsis nevadensis* were used as outgroups.

## References

[CIT0001] Bourguignon T, Lo N, Cameron SL, Šobotník J, Hayashi Y, Shigenobu S, Watanabe D, Roisin Y, Miura T, Evans TA, et al. 2015 The evolutionary history of termites as inferred from 66 mitochondrial genomes. Mol Biol Evol. 32:406–421. 25389205 10.1093/molbev/msu308

[CIT0002] Katoh K, Standley DM 2013 MAFFT multiple sequence alignment software version 7: improvements in performance and usability. Mol Biol Evol. 30:772–780. 23329690 10.1093/molbev/mst010 PMC3603318

[CIT0003] Lanfear R, Frandsen PB, Wright AM, Senfeld T, Calcott B 2016 PartitionFinder2: new methods for selecting partitioned models of evolution for molecular and morphological phylogenetic analyses. Mol Biol Evol. 34:772–773. 10.1093/molbev/msw260 28013191

[CIT0004] Liao Y, Chen H, Lu S, Xie Y, Zhang D 2018 The complete mitochondrial genome of drywood termite, *Incisitermes minor* (Isoptera: Kalotermitidae.). Mitochondrial DNA Part B. 3:324–325. 10.1080/23802359.2017.1422397 PMC7800673 33474159

[CIT0005] Ronquist F, Teslenko M, van der Mark P, Ayres DL, Darling A, Höhna S, Larget B, Liu L, Suchard MA, Huelsenbeck JP 2012 MrBayes 3.2: efficient Bayesian phylogenetic inference and model choice across a large model space. Syst Biol. 61:539–542. 22357727 10.1093/sysbio/sys029 PMC3329765

[CIT0006] Tang C 2015 Discussion on the case of Cryptotermes control. Urban Pest Control. 2:47–47. [in Chinese].

[CIT0007] Zhang D, Gao F, Li WX, Jakovlić I, Zou H, Zhang J, Wang GT 2018 PhyloSuite: an integrated and scalable desktop platform for streamlined molecular sequence data management and evolutionary phylogenetics studies. 2018:489088. 10.1111/1755-0998.13096 31599058

